# Associations of Successful Aging With Socioeconomic Position Across the Life-Course: The West of Scotland Twenty-07 Prospective Cohort Study

**DOI:** 10.1177/0898264316665208

**Published:** 2016-08-30

**Authors:** Elise Whitley, Michaela Benzeval, Frank Popham

**Affiliations:** 1 University of Glasgow, UK; 2 University of Essex, UK

**Keywords:** successful aging, life-course, socioeconomic position, cohort

## Abstract

**Objective:** The aim of this study is to investigate how socioeconomic position (SEP) is associated with multidimensional measures of successful aging (SA), and how this varies and accumulates across the life-course. **Method:** Using data from 1,733 Scottish men and women from two cohorts aged around 57 and 76, respectively, we explored associations of SA, based on the Rowe–Kahn model, with 10 measures of SEP measured in childhood and, distally and proximally, in adulthood. **Results:** Individual SEP associations with SA score were generally consistent across different indicators and life stages: Respondents with the most versus least favorable SEP had two additional positive SA dimensions. There was also a strong association between SA and cumulative SEP based on all 10 measures combined; respondents with the most versus least favorable lifelong SEP had four additional positive SA dimensions. **Conclusion:** SEP advantages/disadvantages act and accumulate across the life-course, resulting in widening socioeconomic inequalities in SA in later life.

## Introduction

Populations around the world are aging (Christensen, Doblhammer, Rau, & Vaupel, 2009). This has important implications for health and economic policies, with a growing proportion of older individuals potentially requiring substantial investment in health and long-term care (Bloom et al., 2015). The notion of successful aging (SA) is therefore a research and policy priority, and identifying determinants of SA is crucial. Early work on SA, as defined by researchers, tended to focus on longevity, absence of disease, and good functioning (Cosco, Prina, Perales, Stephan, & Brayne, 2014a; Depp & Jeste, 2006). However, more recent work has given increasing emphasis to the views of older people themselves (Cosco, Prina, Perales, Stephan, & Brayne, 2013, 2014b; Ferri, James, & Pruchno, 2009; Phelan, Anderson, Lacroix, & Larson, 2004; Reichstadt, Sengupta, Depp, Palinkas, & Jeste, 2010; Tate, Lah, & Cuddy, 2003), who consider these clinical aspects to be less important and are more likely to prioritize social engagement, well-being, and independence (Cosco et al., 2013). This disparity in views is highlighted by a number of studies demonstrating that many older people who consider themselves to be aging successfully do not meet researcher-defined SA criteria (McLaughlin, Jette, & Connell, 2012; Montross et al., 2006; Strawbridge, Wallhagen, & Cohen, 2002; von Faber et al., 2001; Young, Frick, & Phelan, 2009). As a result, SA measures based exclusively on clinical dimensions are unlikely to fully capture the aging experience and, in particular, fail to recognize the resilience and compensatory factors used by many older people, even in the face of considerable physical or cognitive decline (Manning, Carr, & Kail, 2016; P. Martin et al., 2015) . It is therefore important to consider a broader, more multidimensional model of SA.

A number of multidimensional models, encompassing both clinical and nonclinical dimensions, have been proposed (Bowling & Iliffe, 2006, 2011; Young, Fan, Parrish, & Frick, 2009). However, there is a trade-off in this context between the ideal inclusion of dimensions of importance to older people and the more pragmatic need for a model that is easily utilized in different populations. The most commonly used multidimensional model of SA, proposed by Rowe and Kahn (1997), includes six dimensions: absence of disease and disability, good physical and cognitive functioning, and good interpersonal and productive social engagement. The inclusion of social engagement in the Rowe–Kahn model is important as it moves the notion of SA from exclusively clinical measures to encompass individual activity and autonomy, for example, through increased social activity, which in turn may improve functioning and lead to reduced disability (World Health Organization, 2001). Discussion of the extent to which the Rowe and Kahn model captures the views of older people is ongoing (Bowling & Iliffe, 2006, 2011; Ferri et al., 2009; Montross et al., 2006; Phelan et al., 2004; Strawbridge et al., 2002; Young, Fan, et al., 2009; Young, Frick, et al., 2009). However, analyses in older populations have demonstrated strong, positive, cross-sectional associations of Rowe–Kahn SA with well-being (Strawbridge et al., 2002) and self-rated health and life satisfaction (Whitley, Popham, & Benzeval, 2016), and it is of note, in the latter analyses, that self-rated health and life satisfaction were strongly associated with all six dimensions, including interpersonal and productive social engagement, and that associations were consistent across age, gender, occupational socioeconomic position (SEP), and personality.

The Rowe–Kahn model offers a broader perspective on aging, and those authors highlight the role of individual agency in SA (Rowe & Kahn, 1997), that is, the potential for individuals to shape their own aging experience through personal and lifestyle choices. However, critics have suggested that this may be an overly simplistic view (Katz & Calasanti, 2015; Martinson & Berridge, 2015; Stowe & Cooney, 2015) and highlighted that, although “good” health behaviors are often widely understood, opportunities to adopt them are not always equitable and, rather, are constrained by wider societal forces, events, and experiences throughout the life-course. One of the most important factors in this context is SEP with continuing or increasing inequalities in health and health behaviors observed for SEP measured at all stages of the life-course (Isaacs & Schroeder, 2004; Lowry, Kann, Collins, & Kolbe, 1996; Lynch, Kaplan, & Salonen, 1997; Mackenbach, 2012; Marmot et al., 1991). This problem is particularly pertinent in the study of SA, as socioeconomic advantages/disadvantages accumulate throughout life, meaning that disparities between lower and higher SEP individuals will potentially be far greater for SA than outcomes earlier in life.

Lower rates of mortality, morbidity, and disability among individuals with higher SEP are well documented (Adler, Boyce, Chesney, Folkman, & Syme, 1993; Isaacs & Schroeder, 2004; Kunst, Groenhof, Mackenbach, & EU Working Group on Socioeconomic Inequalities in Health, 1998; Marmot et al., 1991). However, SA, particularly as defined by older people, encompasses other, nonclinical dimensions and it is important to understand how SEP shapes the wider aging experience. In practice, less is known about how the full spectrum of SA measures varies according to SEP in older populations (Kuh, Karunananthan, Bergman, & Cooper, 2014; Pongiglione, De Stavola, & Ploubidis, 2015). A handful of existing studies consider SEP associations with Rowe–Kahn SA (Brandt, Deindl, & Hank, 2012; Jang, Choi, & Kim, 2009; Kok, Aartsen, Deeg, & Huisman, 2016; McLaughlin, Connell, Heeringa, Li, & Roberts, 2010; Meng & D’Arcy, 2014; Ng, Broekman, Niti, Gwee, & Kua, 2009; Strawbridge et al., 2002). However, a number of these have included other additional dimensions, for example, well-being or life satisfaction, which limits comparability between study populations. Moreover, several include only a subset of the Rowe–Kahn dimensions and, specifically, most include only a single, combined social engagement variable. As the inclusion of social engagement, both interpersonal and productive, is what distinguishes the Rowe–Kahn from other models, this is an important omission. In addition, existing studies have considered a limited number of SEP measures at just one or two life stages, with childhood and early adulthood particularly underresearched. This is an important limitation as SEP encompasses many dimensions such as education, income, wealth, social status, working conditions, and job security, all acting across the life-course (Braveman et al., 2005; Krieger, Williams, & Moss, 1997). Individual measures have been shown to capture different aspects of SEP and cannot be regarded as simply being markers of the same thing (Benzeval et al., 2014; Braveman et al., 2005). Similarly, it cannot be assumed that the impact of different SEP measures will be the same for all outcomes. For example, in the context of Rowe–Kahn, education would be expected to be particularly strongly associated with cognitive function (Ritchie, Bates, Der, Starr, & Deary, 2013). The timing of SEP measures may also influence associations with SA; for example, occupation and current income might be more important in middle age while pensions and wealth may have a greater impact in retirement (Kawachi, Adler, & Dow, 2010). In addition, it is known that SEP measures mean different things to different groups (Braveman et al., 2005), for example, men and women or younger and older individuals. It is therefore important that investigation of the relationship between SEP and SA be based on multiple measures of SEP, both objective and subjective and covering the full life-course, and that associations are explored in different social groups, for example, defined by gender and age. In addition, recognizing SEP as a lifelong exposure, it is important to establish whether its association with SA is cumulative, to understand at what age associations become apparent and to identify any critical periods when potential interventions might be most effectively introduced.

SA is an important goal in aging populations worldwide, but while aspects of SA are amenable to change through individual choice and behaviors, broader social structures, as captured by SEP, will also affect the aging experience. The aim of our study was therefore to explore associations of SEP throughout the life-course with SA, based on the Rowe–Kahn model, in two large population-based cohorts of men and women, one pre- and one poststatutory retirement age and aging 20 years apart. We have built on previous work by incorporating all six Rowe–Kahn dimensions, including younger-old and older-old cohorts, and exploring individual and cumulative associations with 10 different measures of SEP from across the life-course. These include SEP measured in childhood versus adulthood, objectively versus subjectively, proximally versus distally, and covering occupation, income, and markers of wealth and consumption. Specifically, we consider (a) whether different measures of SEP have different associations with SA and SA dimensions, (b) whether SA associations with cumulative (lifelong) SEP are stronger than associations with individual SEP indicators, (c) whether associations are similar in the younger-old and older-old group, and (d) whether associations differ between men and women.

## Method

The West of Scotland Twenty-07 study is a population-based multiple-cohort study (Benzeval et al., 2009), following three age-cohorts of men and women in the West of Scotland born around 1932, 1952, and 1972. An initial approach was made by Strathclyde Regional Council to 8,266 people of whom 5,184 (63%) agreed to be contacted. Of these, 4,510 (87%) took part in the study. Baseline interviews were conducted in 1987/1988, when the three cohorts were approximately 55, 35, and 15 years old. Respondents were representative of the population of the sampled area (Der, 1998). There were four follow-up waves in 1990/1992, 1995/1997, 2000/2004, and 2007/2008. Ethics approval was gained for each wave from the National Health Service and/or Glasgow University Ethics Committees. Current analyses are based on the two oldest cohorts who aged 20 years apart in differing social and economic contexts. The youngest cohort, aged 15 and therefore preemployment at baseline, was not included, as measures of SEP in the early waves were not comparable with those in the older two cohorts.

### SA Measure

Our SA measure was based on the Rowe–Kahn model, using data from the final wave when respondents were aged around 76 and 57. We have previously presented this measure in detail, and found strong, positive, cross-sectional associations with respondents’ views of their own aging (Whitley et al., 2016). Absence of chronic disease was based on (not having) coronary heart disease (CHD), stroke, chronic obstructive pulmonary disease (COPD), cancer (excluding skin), diabetes, Parkinson’s disease, or serious mental health problems. All other positive dimensions were based on the “best” cohort-specific tertile, acknowledging natural age-related changes in disability and functioning (Weir, Meisner, & Baker, 2010), and allowing differences in social engagement between the working-age and postretirement cohorts. Disability was assessed using Office of Population Censuses and Surveys (now Office for National Statistics (ONS)) disability scores (J. Martin, Meltzer, & Elliot, 1988), which includes questions covering multiple domains, for example, “Can you walk up and down a flight of stairs?”; “Can you raise either arm above your head to reach for something?”; “Can you pick up a mug of coffee?”; “Can you see well enough to read a newspaper?”; “Do you lose control of your bladder at least once a week?”; “Can you use the telephone?” Good physical functioning was defined as 3+ of above median grip strength or Forced Expiratory Volume (FEV_1_), and below median systolic blood pressure or pulse. Cognitive function was based on Alice Heim 4 Test of General Intelligence (Part 1; Heim, 1970). Good interpersonal engagement in both cohorts was defined as 3+ of living with spouse/partner, recent contact with family/friends, and attendance at clubs/classes. Productive engagement was based on work, training, volunteering, child care, supporting others, and group memberships, with a higher cutoff in the 1952 (3+) versus the 1932 (2+) cohort reflecting higher employment rates in this group. SA is most commonly represented in the literature as a dichotomy, with individuals succeeding or failing to meet a set of criteria; in the context of the Rowe–Kahn model, this would mean having a positive outcome for all six dimensions. However, it is increasingly recognized that this may be overly stringent, evidenced by the large number of older people who consider themselves to be aging well but fail to meet these criteria (McLaughlin et al., 2012; Montross et al., 2006; Strawbridge et al., 2002; von Faber et al., 2001; Young, Frick, et al., 2009). SA may therefore be more realistically viewed as a continuum (Bowling, 2007; Bowling & Iliffe, 2011; Whitley et al., 2016; Young, Fan, et al., 2009; Young, Frick, et al., 2009), recognizing the *extent* of success rather than a simple pass/fail, and we use this approach here. SA scores among respondents who survived to the final wave were based on the number of positive dimensions, ranging from 0 (no positive dimensions) to 6 (all six positive dimensions). For the analyses of SA score presented here, respondents who died during follow-up were also included, to avoid biases due to selective mortality, and were given a SA score of −1, representing the least successful outcome. This approach has been used previously in the context of self-rated health (Benzeval, Green, & Leyland, 2011). Results for models excluding respondents who died during follow-up were very similar (not shown), suggesting that associations were not simply due to lower mortality in those with more favorable SEP. Analyses of specific SA dimensions were restricted to respondents who were alive and interviewed in the final wave.

### SEP Measures

We used 10 measures of SEP, based on data from all waves to capture aspects of SEP over different periods of the life-course. Childhood SEP was based on parental occupation, whereas education, from age respondent left school, represents the transition SEP into adulthood (Robertson, Popham, & Benzeval, 2014). Four distal (adult) SEP measures, based on the first wave of data collection, up to 20 years prior to the SA measure, included occupational class, two subjective measures based on respondents’ feelings about their current and future financial situation, and an objective measure of income (net equivalized family income based on the McClements equivalence scale with quintiles defined separately in each cohort; McClements, 1977). Four proximal SEP measures, assessed contemporaneously with the SA measure at the final wave or the wave immediately preceding death, were occupational class, subjective feelings about income, objective income, and a measure of material wealth/consumption from housing tenure and car ownership.

### Statistical Methods

Analyses were based on respondents with complete data for all SA dimensions and SEP measures. One difficulty with analyses based on different SEP measures is that the number and size of categories vary between measures, making direct comparison between their respective associations with SA difficult. We therefore derived an Index of Inequality (Kunst et al., 1998; Mackenbach & Kunst, 1997) for each SEP measure, which puts all measures on the same scale and means that estimates based on these measures are less influenced by extremes in the distribution of respondents in each category. The Index of Inequality is based on the cumulative proportion ranking of the study population and produces a score between 0 and 1 (the lowest and highest possible SEP, respectively) based on the midpoint of the proportion of the population in each category. For example, if the proportion of respondents in a four-category SEP measure is 0.1 (lowest SEP), 0.3, 0.4, and 0.2 (highest SEP), then respondents in the lowest category are assigned its midpoint value of 0.05 (0.1 / 2), and those in subsequent categories are given values of 0.25 (0.1 + (0.3 / 2)), 0.6 (0.1 + 0.3 + (0.4 / 2)), and 0.9 (0.1 + 0.3 + 0.4 + (0.2 / 2))respectively. Using this method, the different SEP measures are all scaled from least to most favorable, on a scale between 0 and 1, and results based on the different Indices of Inequality are therefore comparable. The Slope Index of Inequality (SII) for each SEP measure was obtained by regressing SA score on the corresponding Index of Inequality and compares those with the most versus least favorable SEP. In addition to exploring individual SEP associations, we also considered SEP accumulated across all periods and measures by summing the Indices of Inequality for all 10 SEP measures (resulting in a scale between 0 and 10) and then rescaling to get a score between 0 and 1. This cumulative SII compares respondents with the most versus least favorable SEP accumulated across all periods and measures, with higher rankings given to those with a greater number of favorable SEP measures across the life-course. We also examined SEP associations with individual, binary, SA dimensions in respondents still alive in the final wave using the Relative Index of Inequality (RII), based on logistic regression of SA dimensions on the Indices of Inequality in the same way. Analyses based on all respondents combined were adjusted for gender and age-cohort, while those stratified by gender are adjusted for age-cohort and vice versa. In sensitivity analyses, we repeated analyses with missing values for SA and SEP variables imputed using chained equations (Royston, 2004, 2005a, 2005b), with 20 sets of imputations, and results were very similar to those presented here. For clarity and ease of interpretation, we present unimputed results.

## Results

The 1932 and 1952 cohorts consisted of 1,550 and 1,444 respondents, respectively, of whom 325 (21%) and 362 (25%) were alive but not interviewed in Wave 5, and 369 (24%) and 205 (14%) had missing values for at least one SA dimension (most commonly cognitive function) or SEP measure, leaving 856 and 877 in our analytical sample. Respondents not included in analyses had somewhat lower SA scores and less favorable SEP. Mean (*SD*) age among 1932 and 1952 survivors at Wave 5 was 76.2 (0.6) and 57.1 (0.8), respectively.

Characteristics of the study population are presented in Table 1. There were approximately equal numbers of men and women in the two cohorts, and by the final wave of data collection, 530 (31%) respondents had died, the majority (89%) of deaths occurring in the older cohort. Among those who were still alive, just over half had two or fewer positive SA dimensions and less than 1% had all six positive SA dimensions, the most commonly used (dichotomous) definition of SA.

**Table 1.** Characteristics of the Study Population.

	*n* (%)
Cohort	
1952 cohort	877 (50.6)
1932 cohort	856 (49.4)
Gender	
Male	852 (49.2)
Female	881 (50.8)
Status at Wave 5	
Died	530 (30.6)
Alive and interviewed	1,203 (69.4)
Successful aging score	
1 (died)	530 (30.6)
0 (alive with no positive dimensions)	107 (6.2)
1	234 (13.5)
2	332 (19.2)
3	300 (17.3)
4	170 (9.8)
5	51 (2.9)
6 (alive with all six positive dimensions)	9 (0.5)

**Figure 1.**
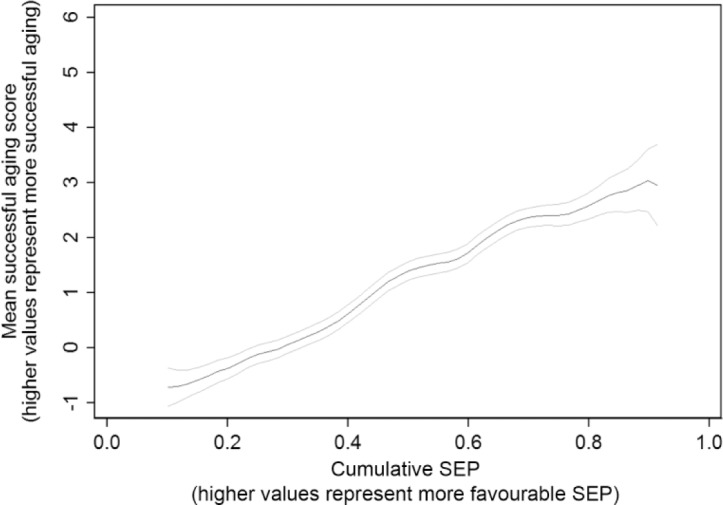
Mean successful aging scores (95% confidence interval) by cumulative SEP (smoothed using Kernel-weighted local polynomials). *Note.* SEP = socioeconomic position.

Table 2 summarizes the 10 SEP measures along with mean SA score in each category. The prevalence of favorable SEP varied across different measures and time points. For example, respondents tended to have more favorable adult than childhood SEP, with around a quarter having a parent with a nonmanual occupation compared with two thirds in a nonmanual occupation themselves at the first or most recent wave. Regardless of the measure, SA scores increased with increasingly favorable SEP. Mean SA scores are plotted against cumulative SEP in Figure 1; scores increased steadily with more favorable cumulative SEP with no evidence of any threshold or saturation effects.

**Table 2.** Successful Aging Score by Socioeconomic Position Across the Life-Course.

	*n* (%)^a^	*M* (*SD*) successful aging score
Childhood socioeconomic position		
Parental occupation		
V	231 (13.3)	0.6 (1.8)
IV	318 (18.4)	1.0 (1.8)
III manual	728 (42.0)	1.2 (1.9)
III non manual	140 (8.1)	1.9 (1.8)
II	245 (14.1)	2.1 (1.8)
I	71 (4.1)	2.3 (2.0)
Age left education		
At or before leaving age	1,008 (58.2)	0.8 (1.7)
Beyond leaving age	518 (29.9)	1.9 (1.9)
Full school education	207 (11.9)	2.5 (1.6)
Distal (Wave 1) socioeconomic position		
Own occupation		
V	74 (4.3)	0.0 (1.4)
IV	216 (12.5)	0.4 (1.6)
III manual	352 (20.3)	0.6 (1.6)
III non-manual	402 (23.2)	1.4 (1.7)
II	525 (30.3)	2.0 (1.9)
I	164 (9.5)	2.3 (1.9)
Ease of making ends meet		
Difficult	418 (24.1)	0.3 (1.6)
Moderate	878 (50.7)	1.3 (1.8)
Easy	437 (25.2)	2.3 (1.8)
Confidence in future finances		
Very insecure	394 (22.7)	0.1 (1.5)
Fairly insecure	176 (10.2)	1.4 (1.6)
Fairly secure	997 (57.5)	1.7 (1.9)
Very secure	166 (9.6)	1.7 (1.9)
Income^b^		
1 (lowest quintile)	309 (17.8)	0.5 (1.6)
2	338 (19.5)	0.9 (1.8)
3	339 (19.6)	1.3 (1.8)
4	363 (21.0)	1.6 (1.9)
5 (highest quintile)	384 (22.2)	2.0 (2.0)
Proximal (most recent) socioeconomic position		
Own occupation		
V	90 (5.2)	0.2 (1.5)
IV	229 (13.2)	0.4 (1.6)
III manual	326 (18.8)	0.5 (1.6)
III non-manual	359 (20.7)	1.3 (1.8)
II	559 (32.3)	2.0 (1.8)
I	170 (9.8)	2.4 (1.8)
Feelings about income		
1 (most negative)	68 (3.9)	−0.4 (1.1)
2	59 (3.4)	−0.1 (1.3)
3	95 (5.5)	0.3 (1.6)
4	212 (12.2)	0.7 (1.7)
5	519 (30.0)	1.2 (1.8)
6	502 (29.0)	1.9 (1.8)
7 (most positive)	278 (16.0)	1.9 (1.9)
Income^b^		
1 (lowest quintile)	260 (15.0)	0.1 (1.5)
2	322 (18.6)	0.8 (1.8)
3	368 (21.2)	1.3 (1.8)
4	377 (21.8)	1.8 (1.8)
5 (highest quintile)	406 (23.4)	2.0 (1.9)
Housing tenure and car ownership		
No tenure/no car	352 (20.3)	−0.1 (1.3)
No tenure/car	175 (10.1)	0.4 (1.8)
Tenure/no car	227 (13.1)	1.0 (1.7)
Tenure/car	979 (56.5)	2.0 (1.8)

a Analytical sample based on complete cases (*N* = 1,733).

b Income quintiles calculated separately for the two cohorts to allow for differences in economic status, in particular pre- and postretirement.

**Figure 2.**
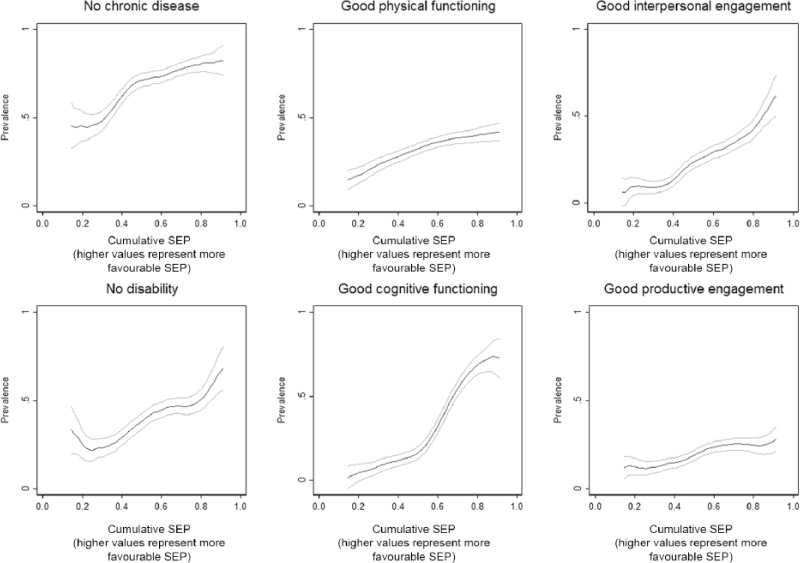
Prevalence (95% confidence interval) of positive successful aging dimensions by cumulative SEP (smoothed using Kernel-weighted local polynomials). *Note.* SEP = socioeconomic position.

SIIs (95% confidence interval [CI]) from least squares regression models for each SEP measure are presented in Table 3. Results for all respondents combined are shown in the first column. The smallest SII was observed for parental occupation, with respondents with the most favorable parental occupation having SA scores around 1.27 [0.99, 1.55] higher than those with least favorable parental occupation. SIIs for education, all distal SEP measures, and proximal SEP measures other than housing tenure and car ownership were very similar; SA scores in respondents with the most versus least favorable SEP were around 2 points higher, the equivalent of two additional positive SA dimensions. The largest individual SII was observed for (proximal) housing tenure and car ownership (2.66 [2.36, 2.96]). SA associations with different SEP measures, although somewhat attenuated, were still evident after adjusting for the other measures (e.g., SII for proximal tenure/car ownership adjusted for all other SEP measures = 1.45 [1.10, 1.80]). The association of SA score with cumulative (lifelong) SEP was stronger than for any individual SEP indicators, with SA scores for respondents with the most favorable cumulative SEP 4.38 [3.98, 4.78] higher than respondents with the least favorable cumulative SEP. Analyses stratified by gender and age-cohort were broadly similar with marginally stronger associations of SA with age left school in females and with childhood SEP and (distal) ease of making ends meet in the older cohort The prevalence of individual positive SA dimensions among those alive in Wave 5 is plotted against cumulative SEP in Figure 2 (all respondents combined). Although the overall prevalences of different SA dimensions varied, all increased with increasing cumulative SEP. RIIs (95% CI) for individual and cumulative SEP associations with positive SA dimensions are presented in Table 4. All SEP–SA dimension associations were positive although the strength of associations varied, with generally weaker associations with parental occupation and stronger associations with proximal tenure and car ownership, particularly for good interpersonal engagement. The exception to this was good cognitive function, where the strongest associations were with education, and adult occupation and income. The most marked associations with individual and cumulative SEP were observed for good cognitive function (RII, 95% CI for cumulative SEP = 52.67 [33.01, 84.05]), interpersonal engagement (15.00 [8.11, 27.71]), and productive engagement (5.21 [2.63, 10.30]).

**Table 3.** Difference^a^ (95% Confidence Interval) in Successful Aging Score According to Most Versus Least Favorable Individual and Cumulative Socioeconomic Position (Based on Indices of Inequality).

	All respondents (*N* = 1,733)	Stratified by gender		Stratified by age-cohort	
		Females (*n* = 881)	Males (*n* = 852)	1952 cohort (*n* = 877)	1932 cohort (*n* = 856)
Childhood socioeconomic position					
Parental occupational class	1.27 [0.99, 1.55]	1.15 [0.79, 1.52]	1.39 [0.96, 1.82]	1.00 [0.66, 1.34]	1.57 [1.12, 2.03]
Age left school	1.94 [1.64, 2.25]	2.23 [1.82, 2.63]	1.65 [1.19, 2.10]	1.75 [1.40, 2.10]	2.19 [1.67, 2.71]
Distal (Wave 1) socioeconomic position					
Own occupational class	2.03 [1.76, 2.30]	1.98 [1.61, 2.34]	2.08 [1.68, 2.47]	1.98 [1.64, 2.32]	2.07 [1.66, 2.49]
Ease of making ends meet	1.70 [1.40, 2.01]	1.64 [1.23, 2.05]	1.77 [1.32, 2.22]	1.27 [0.90, 1.65]	2.18 [1.71, 2.66]
Confidence in future finances	1.73 [1.43, 2.03]	1.81 [1.41, 2.21]	1.65 [1.20, 2.11]	1.81 [1.34, 2.29]	1.69 [1.29, 2.10]
Income	1.76 [1.50, 2.03]	1.62 [1.26, 1.98]	1.90 [1.50, 2.30]	1.85 [1.51, 2.19]	1.67 [1.25, 2.09]
Proximal (most recent) socioeconomic position					
Own occupational class	1.91 [1.63, 2.18]	1.93 [1.56, 2.30]	1.87 [1.46, 2.28]	1.88 [1.54, 2.23]	1.92 [1.48, 2.35]
Feelings about income	1.91 [1.63, 2.18]	1.87 [1.51, 2.23]	1.93 [1.52, 2.35]	1.80 [1.44, 2.15]	1.99 [1.58, 2.41]
Income	2.07 [1.79, 2.34]	2.10 [1.74, 2.46]	2.02 [1.61, 2.43]	2.12 [1.77, 2.47]	2.00 [1.59, 2.42]
Housing tenure and car ownership	2.66 [2.36, 2.96]	2.44 [2.03, 2.85]	2.91 [2.47, 3.35]	2.65 [2.24, 3.06]	2.70 [2.27, 3.13]

Cumulative socioeconomic position	4.38 [3.98, 4.78]	4.46 [3.91, 5.00]	4.30 [3.70, 4.89]	4.27 [3.75, 4.80]	4.46 [3.84, 5.07]

a Adjusted for age-cohort and gender (all respondents), age-cohort (analyses stratified by gender), and gender (analyses stratified by age-cohort).

**Table 4.** Risk Ratio^a^ (95% Confidence Interval) for Positive Successful Aging Dimension According to Most Versus Least Favorable Individual and Cumulative Socioeconomic Position (Based on Indices of Inequality).

	No chronic disease	No disability	Good physical functioning	Good cognitive functioning	Good interpersonal engagement	Good productive engagement
Childhood socioeconomic position						
Parental occupation	1.10 [0.98, 1.24]	1.13 [0.90, 1.42]	1.30 [0.98, 1.72]	4.17 [3.06, 5.67]	1.92 [1.35, 2.73]	1.38 [0.91, 2.11]
Age left School	1.21 [1.07, 1.37]	1.20 [0.95, 1.52]	1.39 [1.04, 1.87]	10.28 [7.50, 14.08]	2.62 [1.84, 3.72]	2.12 [1.38, 3.25]
Distal (Wave 1) socioeconomic position						
Own occupation	1.17 [1.03, 1.32]	1.59 [1.24, 2.03]	1.69 [1.27, 2.26]	9.48 [6.67, 13.47]	3.79 [2.56, 5.61]	2.03 [1.32, 3.13]
Make ends meet	1.22 [1.07, 1.38]	1.59 [1.24, 2.05]	1.61 [1.18, 2.18]	3.57 [2.50, 5.11]	2.44 [1.63, 3.65]	2.68 [1.69, 4.23]
Future finances	1.37 [1.18, 1.59]	1.56 [1.18, 2.07]	1.76 [1.25, 2.49]	2.91 [2.03, 4.17]	2.74 [1.83, 4.09]	1.67 [1.06, 2.62]
Income	1.29 [1.15, 1.46]	1.49 [1.17, 1.89]	1.69 [1.26, 2.22]	7.37 [5.07, 10.73]	2.56 [1.79, 3.67]	1.44 [0.97, 2.13]
Proximal (most recent) socioeconomic position						
Own occupation	1.26 [1.12, 1.43]	1.46 [1.14, 1.86]	1.40 [1.06, 1.86]	7.03 [5.02, 9.85]	3.21 [2.20, 4.69]	3.21 [2.07, 4.98]
Finance feelings	1.32 [1.16, 1.50]	1.83 [1.43, 2.34]	1.42 [1.06, 1.92]	3.05 [2.21, 4.21]	2.33 [1.63, 3.33]	1.64 [1.08, 2.49]
Income	1.25 [1.10, 1.42]	1.35 [1.04, 1.75]	1.76 [1.28, 2.42]	5.70 [3.82, 8.51]	2.59 [1.75, 3.83]	1.60 [1.04, 2.47]
Car/tenure	1.42 [1.21, 1.68]	1.85 [1.31, 2.61]	3.00 [1.85, 4.86]	4.74 [2.75, 8.17]	16.14 [5.86, 44.49]	3.03 [1.59, 5.78]

Cumulative socioeconomic position	1.73 [1.41, 2.11]	2.62 [1.78, 3.90]	2.93 [1.85, 4.65]	52.67 [33.01, 84.05]	15.00 [8.11, 27.71]	5.21 [2.63, 10.30]

a Adjusted for age-cohort and gender.

## Discussion

Although in principle different aspects of SA are under individual control, in practice wider factors such as SEP will also influence the aging process. Mortality and morbidity are known to be lower in individuals with more favorable SEP (Adler et al., 1993; Isaacs & Schroeder, 2004; Kunst et al., 1998; Marmot et al., 1991) but, in comparison, very little is known about the social patterning of multidimensional measures of SA and, in particular, how associations with SEP change and accumulate throughout the life-course. Previous studies of SEP associations with Rowe–Kahn SA (Brandt et al., 2012; Jang et al., 2009; Kok et al., 2016; McLaughlin et al., 2010; Meng & D’Arcy, 2014; Ng et al., 2009; Strawbridge et al., 2002) have reported positive associations with most, although not all (Meng & D’Arcy, 2014), SEP measures. However, utilization of the Rowe–Kahn model in these studies varies, with some dimensions missing or combined and/or extra dimensions added, making direct comparisons difficult. In addition, a limited number of SEP measures have been considered, and they have not generally covered the range of life stages or SEP dimensions included here. Previous work has also considered SA as a dichotomy, which may be too restrictive to realistically represent the views of older people (McLaughlin et al., 2012; Montross et al., 2006; Strawbridge et al., 2002; von Faber et al., 2001; Young, Frick, et al., 2009). We have extended previous work, using data from two large population-based age-cohorts of men and women. The inclusion of two age-cohorts has allowed consideration of early SEP associations with SA in a younger-old group not widely considered elsewhere and also the comparison of SEP–SA associations in two cohorts aging 20 years apart in different economic contexts, for example, in terms of the increasing female workforce. We have used all six Rowe–Kahn dimensions to derive an (continuous) SA score that agrees well with respondents’ own view of their aging (Whitley et al., 2016) and explored individual and cumulative associations with 10 different measures of SEP, covering objective and subjective assessments of education, employment, income, and wealth, in childhood and both distal and proximal adulthood.

Although there are many advantages to our analyses, there are also a number of limitations to be considered. Analyses were based on a subgroup of the original cohort, potentially limiting the generalizability of our findings, although results from analyses based on the full cohorts with imputed data were very similar to those presented here. We avoided potential bias due to selective mortality by including respondents who died during follow-up. However, we also considered the possibility that associations were driven by excess mortality in those with less favorable SEP by repeating our analyses excluding those who had died, and the same pattern of results was observed. Finally, although our childhood and distal adult measures of SEP clearly predate the measurement of SA, there is a possibility that associations with proximal SEP, measured concurrently with SA, may be due to static or downward social mobility in respondents in poorer health. However, it is of note that where SEP measures were available both proximally and distally (up to 20 years previously), their associations with SA were very similar in magnitude, suggesting that this was not the case.

All SEP indicators were positively associated with overall SA score, and the magnitude of associations at different life stages was generally similar, consistent with SEP acting throughout the life-course rather than at any specific critical or current time point. Associations remained after mutual adjustment, suggesting that variables were accessing different aspects of SEP at different time points and underlining the importance of considering multiple indicators of SEP at different life stages (Braveman et al., 2005). Weaker associations with parental occupation may indicate a more influential role of adult versus childhood SEP or, perhaps, reflect the greater error inherent in retrospective measurement of childhood experiences (Batty, Lawlor, Macintyre, Clark, & Leon, 2005). Although SEP–SA associations were broadly similar in men and women and in the two age-cohorts, small differences were suggestive of a relatively weaker impact of early life SEP in men and younger-old individuals. However, it is of note that the majority of SEP–SA associations were similar in both age-cohorts, highlighting that the impact of SEP is apparent even in the early stages of aging. All SEP measures used here will, to some extent, reflect past experiences, and this is particularly true of proximal wealth/consumption, based on housing tenure and car ownership, which might explain its stronger association with SA. Alternatively, home and, in particular, car ownership may increase opportunities for social engagement (Curl, Proulx, Stowe, & Cooney, 2015) and, consistent with this, in our data, proximal wealth was strikingly strongly associated with interpersonal engagement.

SEP indicators were also positively associated with all six individual SA dimensions. Lower rates of disease and disability and better physical and cognitive functioning in individuals with more favorable SEP have been reported previously. Associations of cognition with education, occupation, and income were particularly strong, possibly reflecting their bidirectionality. It is noteworthy that social engagement dimensions, which are often omitted from SA models, were also strongly associated with all aspects of SEP; indeed, associations with interpersonal engagement were consistently stronger than those for disease, disability, and physical functioning. Associations of SEP with interpersonal and productive engagement are particularly worthy of further investigation as they represent dimensions of considerable importance to older people (Cosco et al., 2013, 2014b; Phelan et al., 2004; Reichstadt et al., 2010) that may be amenable to change through appropriate interventions, for example, programs promoting volunteering and community involvement or schemes that widen access to free public transport such as the National Concessionary Travel Scheme for older and disabled people introduced in Scotland in 2006. In addition, the maintenance of active interpersonal and productive engagement in old age has major health and economic implications in the context of increasingly aging populations (Bloom et al., 2015), and it is important that factors contributing to or hindering its achievement be recognized and understood.

We also observed striking trends of increasing overall SA score and individual positive SA dimension prevalences with increasingly favorable lifetime (cumulative) SEP. These results present a stark contrast, with individuals with the most favorable lifetime SEP having four or more positive SA dimensions than individuals with the most unfavorable lifetime SEP. It is also notable that SEP–SA score associations in the younger-old cohort (aged ~57) were very similar to those for the older-old cohort (aged ~76), suggesting that SEP influences on SA are apparent from a relatively early age, both pre- and postretirement, and highlighting the potential benefits of early intervention. Further work is needed to fully understand the mechanisms underlying these results, for example, considering the role of health behaviors or housing and neighborhood characteristics, with a view to informing policies and interventions aimed at reducing these marked inequalities in the aging experience.

## Conclusion

While individual agency has a role in SA, this is an overly simplistic view that fails to recognize the constraints placed on individuals by wider social forces. Our results demonstrate that associations of socioeconomic advantage/disadvantage with SA are observed at all life stages and accumulate across the life-course in men and women and in different age-cohorts. In the context of aging populations worldwide, further work to understand the mechanisms underlying these associations and to identify potential interventions and intervention points is a priority.

## Summary

- Populations are aging worldwide, and identifying determinants of “successful aging” (SA; good physical, mental, and cognitive health and continuing social and economic activity among older people) is a research and policy priority.
- Although some aspects of SA are potentially within the control of individuals, for example, through lifestyle choices, opportunities to choose and adopt advantageous behaviors are not equal across all socioeconomic groups. Little is known about how socioeconomic position (SEP) is associated with multidimensional measures of SA and, in particular, how this varies and accumulates across the life-course.
- Individuals with a more favorable SEP, based on 10 distinct indicators from childhood, and early and later adulthood, have more positive SA dimensions than those with a less favorable position, and this association is cumulative across the life-course.
- There are marked inequalities in the aging experience of individuals according to SEP observed at all life stages and accumulated across the life-course. In the context of aging populations worldwide, further work to understand the mechanisms underlying these associations and to identify potential interventions and intervention points is a priority.
